# Data from mass spectrometry, NMR spectra, GC–MS of fatty acid esters produced by *Lasiodiplodia theobromae*

**DOI:** 10.1016/j.dib.2016.05.003

**Published:** 2016-05-09

**Authors:** Carla C. Uranga, Joris Beld, Anthony Mrse, Iván Córdova-Guerrero, Michael D. Burkart, Rufina Hernández-Martínez

**Affiliations:** a Centro de Investigación Científica y de Educación Superior de Ensenada (CICESE), Carretera Ensenada-Tijuana 3918, Zona Playitas, 22860 Ensenada, B.C., Mexico; b University of California, Department of Chemistry and Biochemistry, 9500 Gilman Dr., La Jolla, San Diego, CA 92093-0358, USA; c Universidad Autónoma de Baja California (UABC), Calzada Universidad 14418 Parque Industrial Internacional Tijuana, Tijuana, 22390 B.C., Mexico

**Keywords:** NMR, GC–MS, Fatty acid esters, Ethyl stearate, Ethyl linoleate, Growth inhibition, Growth induction

## Abstract

The data described herein is related to the article with the title “Fatty acid esters produced by *Lasiodiplodia theobromae* function as growth regulators in tobacco seedlings” C.C. Uranga, J. Beld, A. Mrse, I. Cordova-Guerrero, M.D. Burkart, R. Hernandez-Martinez (2016) [1]. Data includes nuclear magnetic resonance spectroscopy and GC–MS data used for the identification and characterization of fatty acid esters produced by *L. theobromae*. GC–MS traces are also shown for incubations in defined substrate, consisting in Vogel׳s salts supplemented with either 5% grapeseed oil or 5% glucose, the two combined, or 5% fructose. Traces for incubations in the combination of 5% grapeseed oil and 5% glucose for different fungal species are also included. Images of mycelium morphology when grown in 5% glucose with or without 5% grapeseed oil are shown due to the stark difference in mycelial pigmentation in the presence of triglycerides. High concentration gradient data for the plant model *Nicotiana tabacum* germinated in ethyl stearate (SAEE) and ethyl linoleate (LAEE) is included to show the transition between growth inhibition and growth induction in *N. tabacum* by these compounds.

Specifications Table

**Table:** 

Subject area	Microbial biochemistry.
More specific subject area	Fatty acid metabolism by plant fungal pathogens.
Type of data	Mass spectrometry data, NMR spectra, GC–MS chromatograms, photography, microscopy, *N. tabacum* morphology and measurements of early growth in LAEE and SAEE concentration gradients.
How data was acquired	High resolution mass spectrometry: Agilent 6230 ESI-TOF MS. NMR: Varian 500 MHz instrument equipped with an XSens 2-channel NMR cold probe. GC–MS: Agilent 7890A GC system, connected to a 5975C VL MSD quadrupole MS (EI) mass spectroscopy. Olympus stereo microscope (SZX12).
Data format	Analyzed
Experimental factors	Material was purified with silica gel, HPLC and TLC for HR-MS and NMR analysis, followed by GC–MS. Carbon substrates were defined and simplified and subjected to further GC–MS.
Experimental features	Fungal samples were lyophilized, then extracted via a modified Folch extraction using 1:1 v/v dichloromethane and methanol along with 0.01% BHT as an antioxidant.
Data source location	University of California, San Diego, USA and CICESE, Ensenada, Mexico
Data accessibility	All relevant data is provided.

Value of the data

• This is the first report of fatty acid esters naturally produced by *Lasiodiplodia theobromae* and the other fungal species studied.
• Lipases from *L. theobromae* and *Neofusicoccum parvum* have broad substrate specificity that may be of interest for further characterization and potential biotechnological uses.
• Many of the fatty acid esters are novel for phytopathogenic fungi and might open exciting new research areas in fungal lipidomics and plant pathology.

## 1 Data

The data being shared consists in NMR spectra, as well as high-resolution mass spectrometry spectra and gas chromatography–mass spectrometry chromatograms used to identify fatty acid esters from the phytopathogenic fungus, *L. theobromae*. Other fungi such as *Neofusicoccum parvum, Fusarium oxysporum* f.sp. *lycopersici* and *Trichoderma asperellum* were also studied for comparison. Images of mycelial morphology for *L. theobromae* in different carbon sources are shown. Effects of fatty acid esters produced by *L. theobromae* in *N. tabacum* morphology are included. Concentration gradients for the most physiologically active compounds, ethyl stearate (SAEE) and ethyl linoleate (LAEE) are also shown.

## 2 Experimental design, materials and methods

**Fig. 1:**
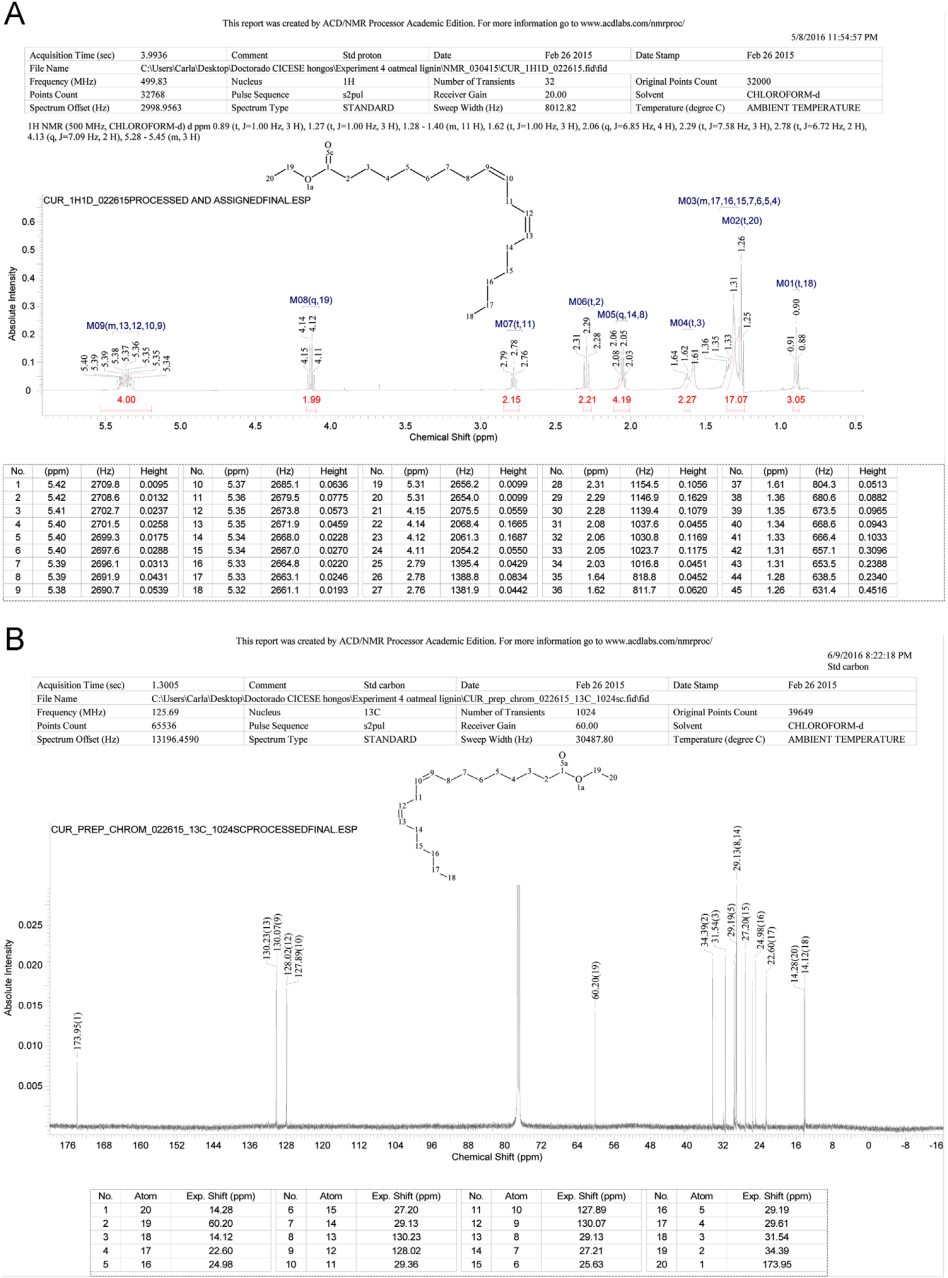
Analysis of the compound isolated from *Lasiodiplodia theobromae*. A: ^1^H NMR spectrum. B: ^13^C NMR spectrum identified as linoleic acid ethyl ester (LAEE).

**Fig. 2:**
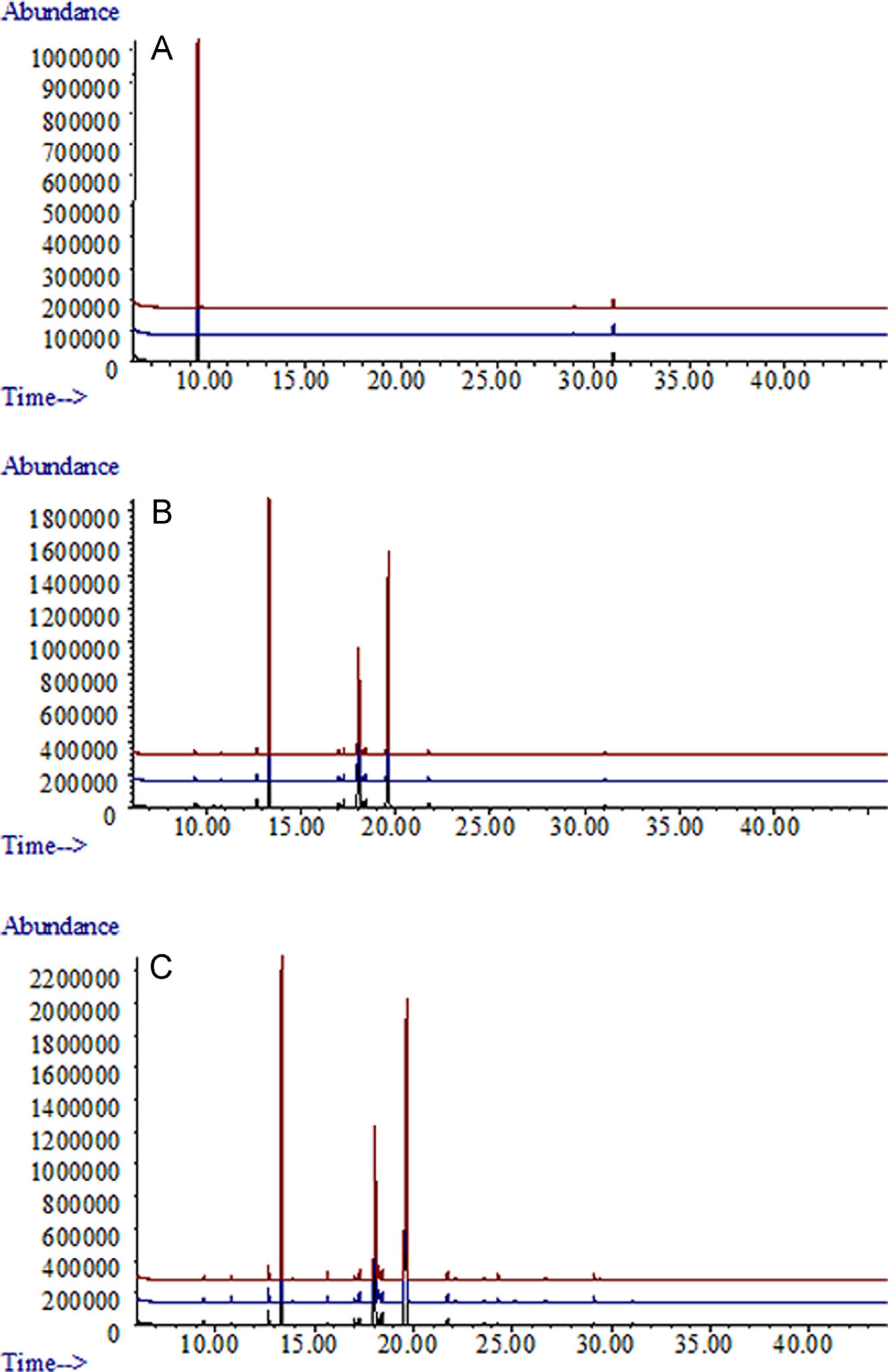
Overview of GC/MS traces from *Lasiodiplodia theobromae* strains incubated in oatmeal. A: Three replicates of negative control (oatmeal only). B: Three replicates of strain UCD256Ma. C: Three replicates of strain MXL28.

**Fig. 3:**
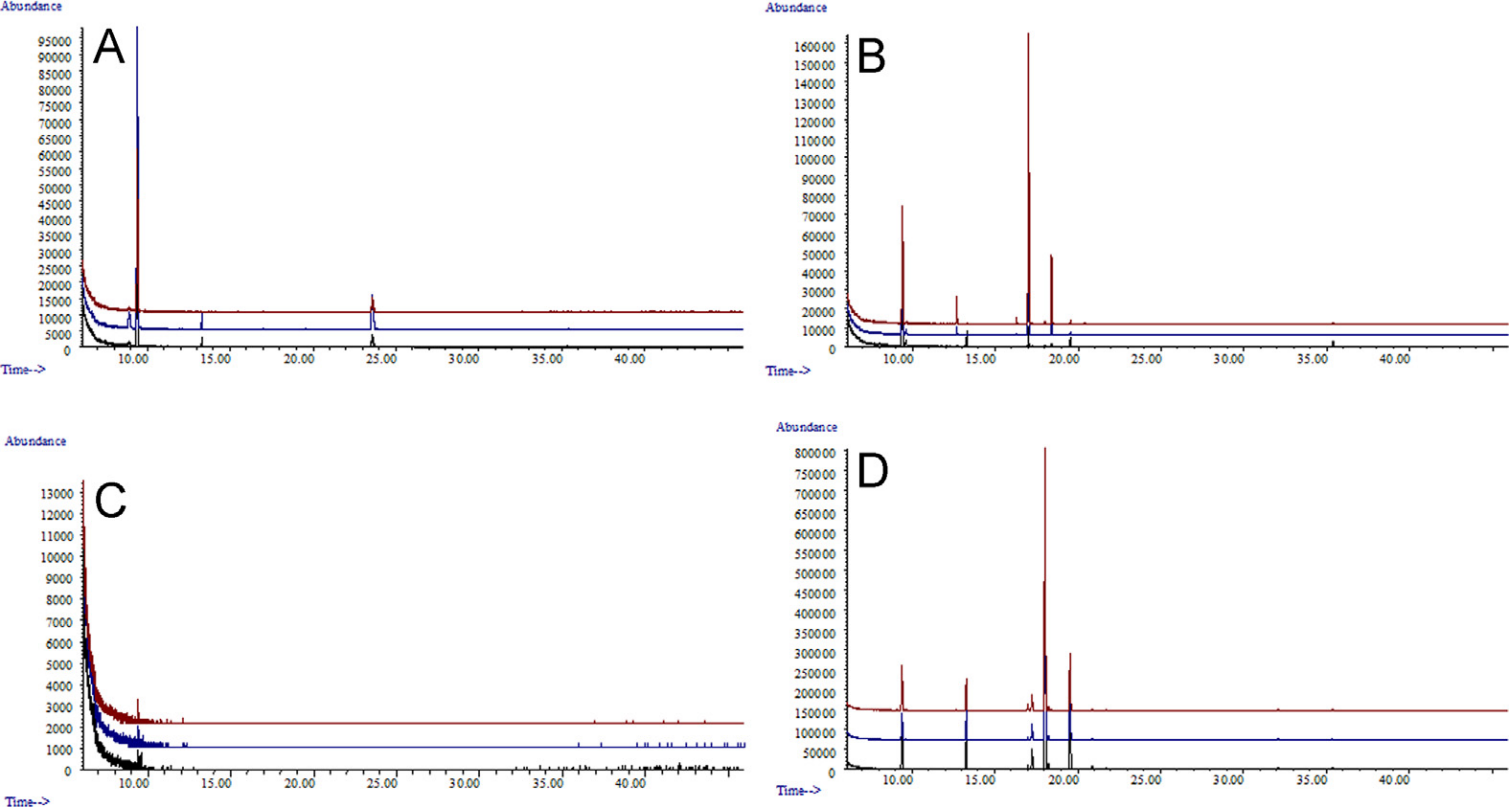
GC/MS traces of *Lasiodiplodia theobromae* (UCD256Ma) incubated in Vogel׳s salts with A; 5% glucose. B; 5% grapeseed oil. C; 5% fructose. D; a combination of 5% glucose+5% grapeseed oil as carbon sources.

**Fig. 4:**
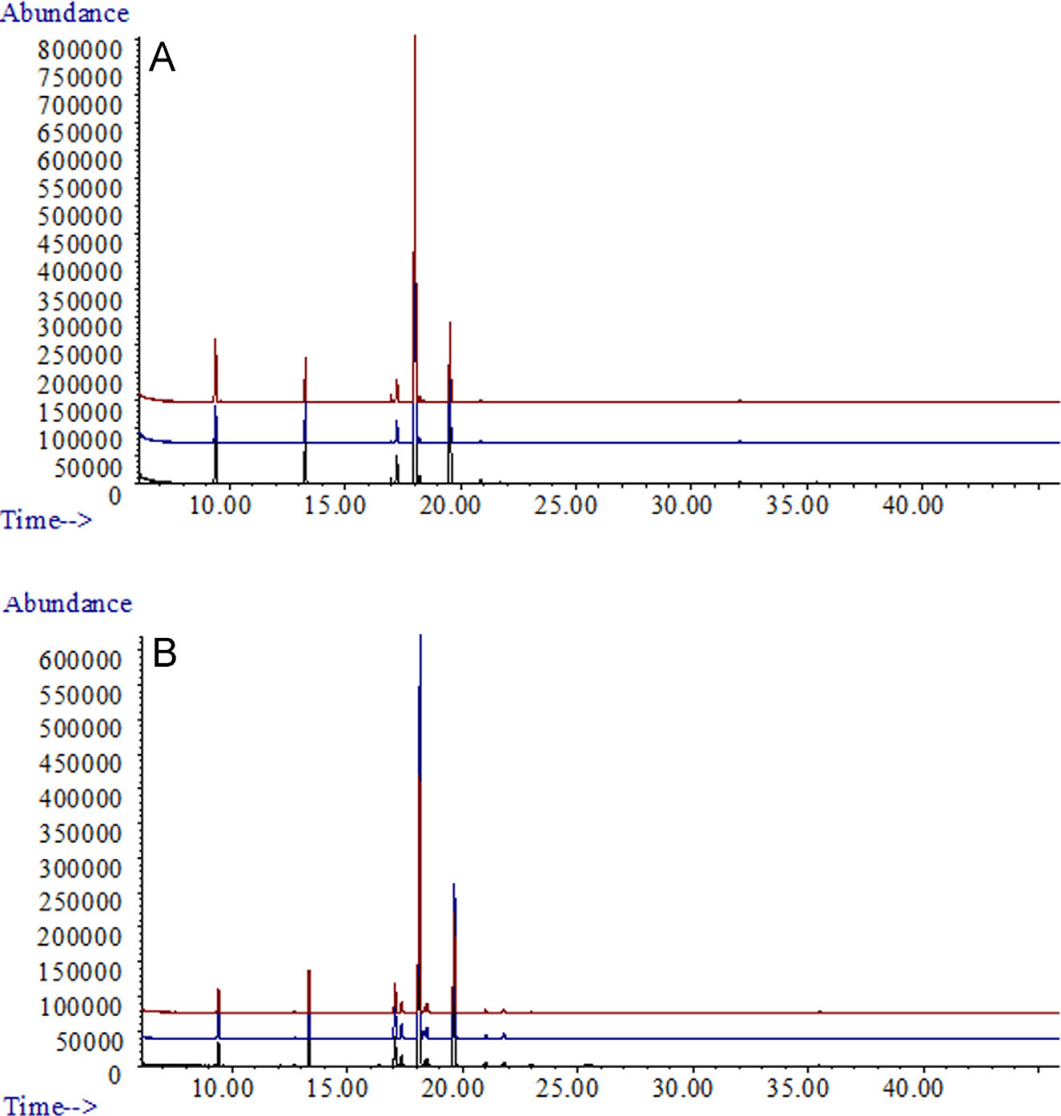
GC/MS traces of A; *Lasiodiplodia theobromae* (UCD256Ma) and B; *Neofusicoccum parvum* (UCD646So). Both were grown in 5% glucose+5% grapeseed oil combined.

**Fig. 5:**
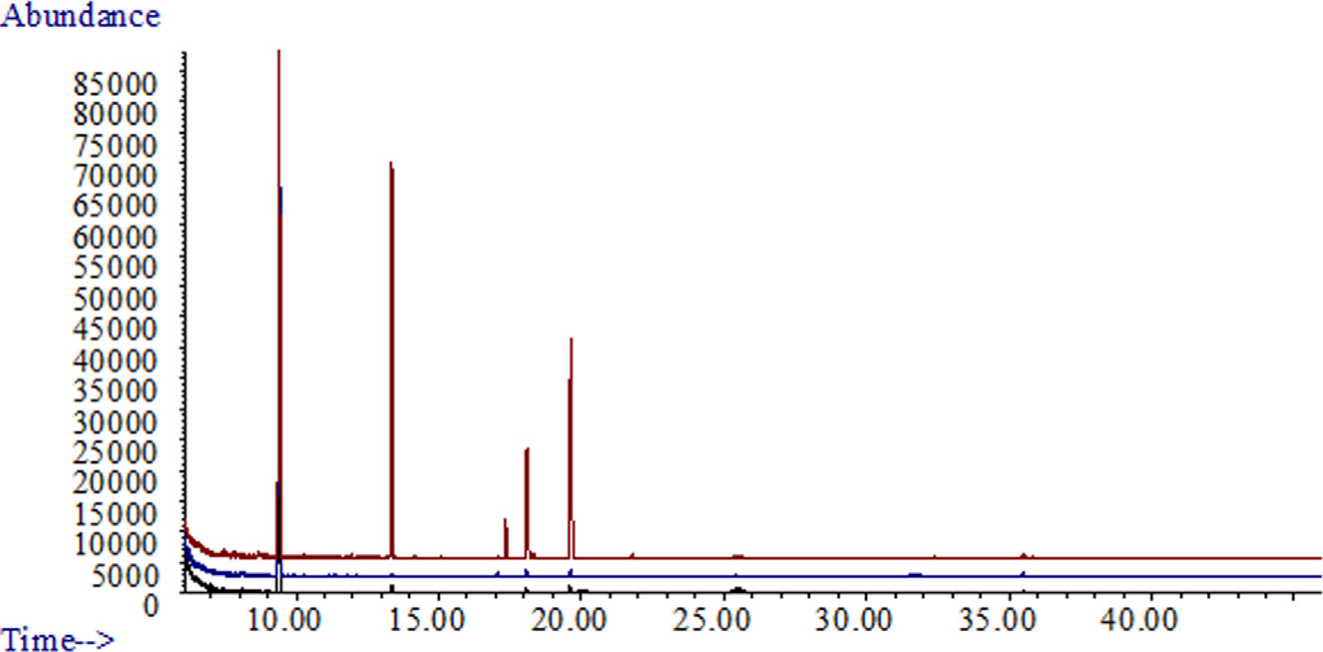
GC/MS traces of a soil borne pathogen *Fusarium oxysporum* f. sp. *lycopersici* incubated in Vogel׳s salts with both 5% glucose and 5% grapeseed oil.

**Fig. 6:**
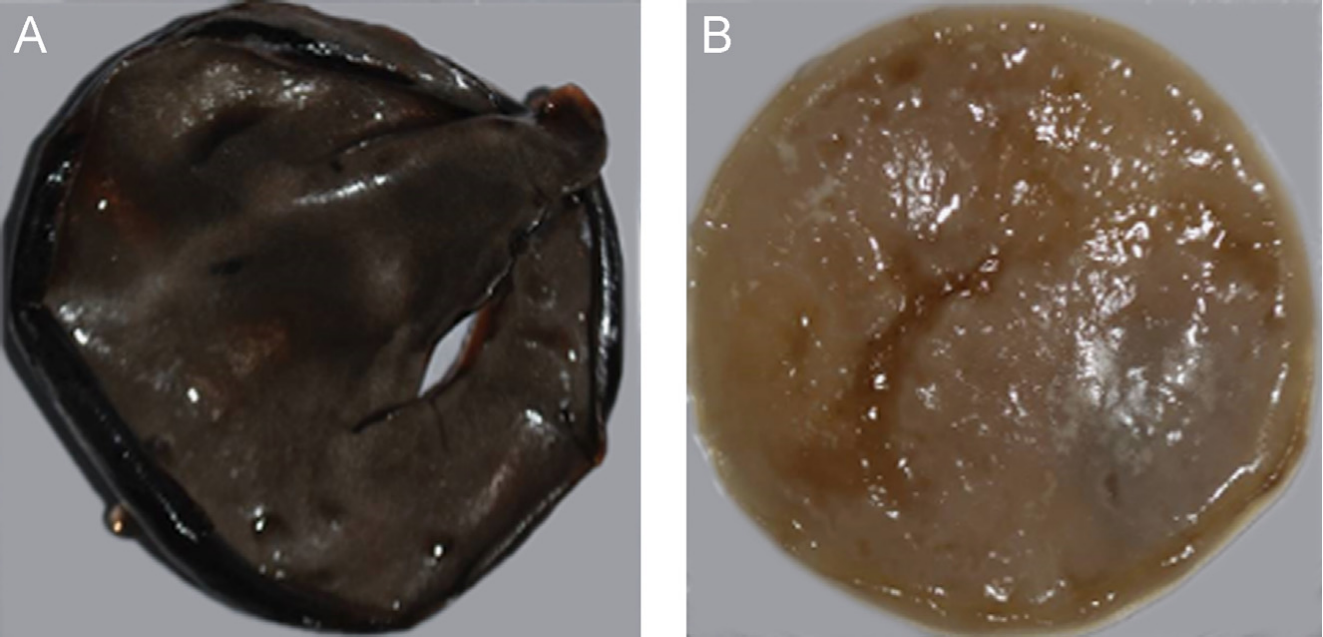
Morphology of *Lasiodiplodia theobromae* (UCD256Ma). A: *Lasiodiplodia theobromae* incubated in 5% glucose in Vogel׳s salts. B: *Lasiodiplodia theobromae* incubated in 5% glucose and 5% grapeseed oil in Vogel׳s salts.

**Fig. 7:**
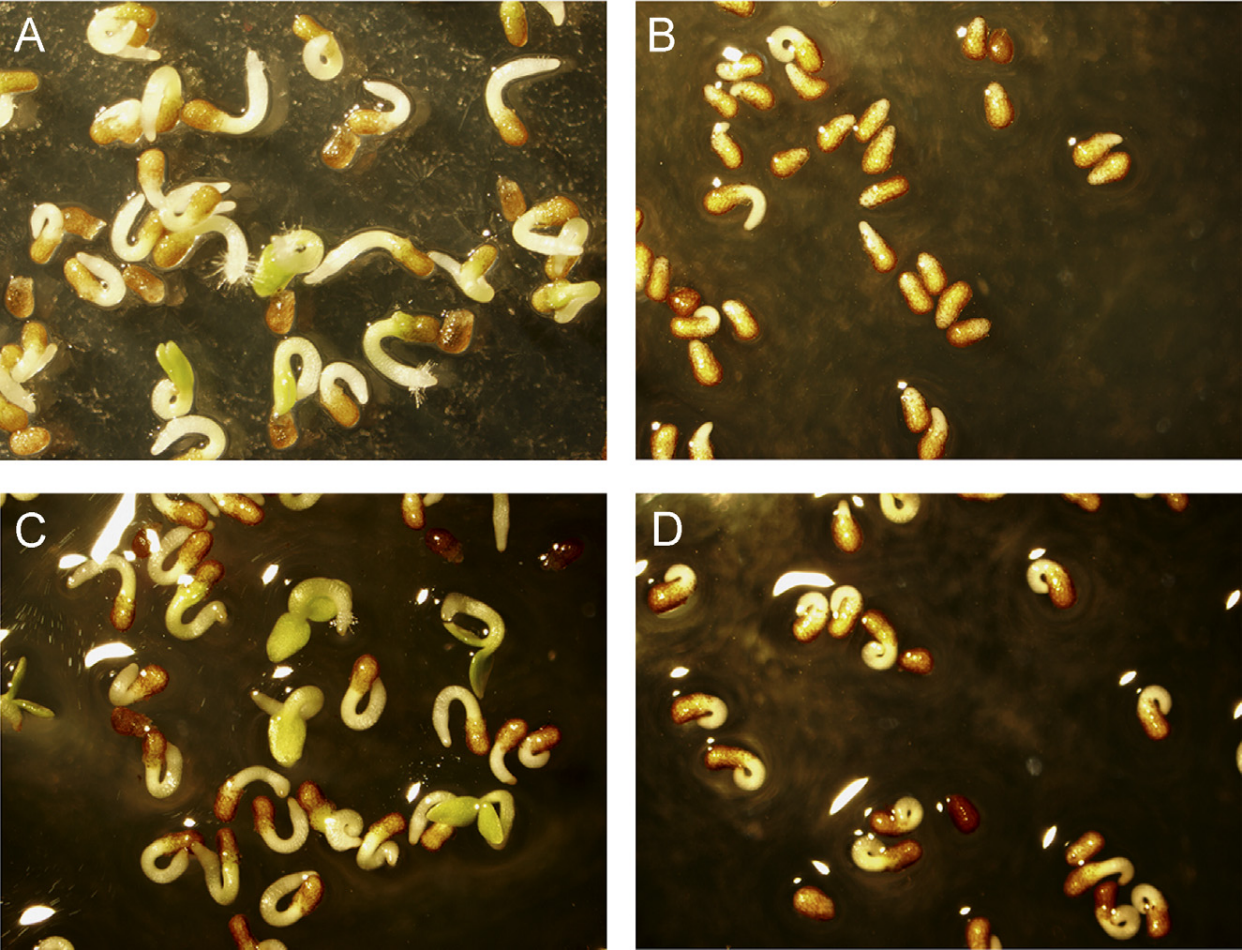
Seed germination in *Nicotiana tabacum* exposed to 100 μg/mL free palmitate, palmitate ethyl ester or linoleate ethyl ester emulsified with 0.08% kolliphor P-188 in Murashige and Skoog salts with Gamborg vitamins, supplemented with 3% sucrose, and 0.4% PPM. A: negative control. B: 0.1 mg/mL LAEE. C: 0.1 mg/mL PA. D: 0.1 mg/mL PAEE.

**Fig. 8:**
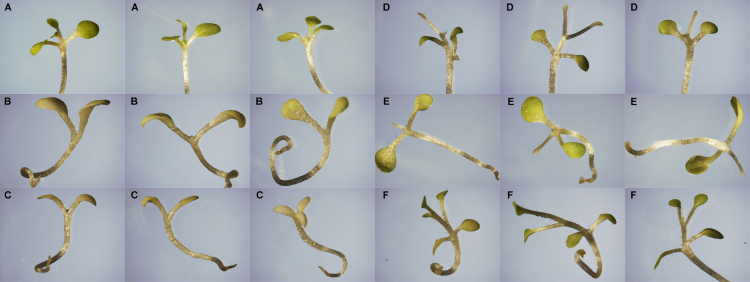
Morphology of *Nicotiana tabacum* germinated in FAE and grown 45 days in Murashige–Skoog+3% sucrose. A; three biological replicates of *N. tabacum* without FAE. B; 0.2 mg/mL PAEE. C; 0.2 mg/mL LAEE. D; 0.2 mg/mL SAEE. E; 3.13 μg/mL SAEE. F; 0.2 mg/mL crude extract of *Lasiodiplodia theobromae* incubated in 5% grapeseed oil+5% glucose.

**Fig. 9:**
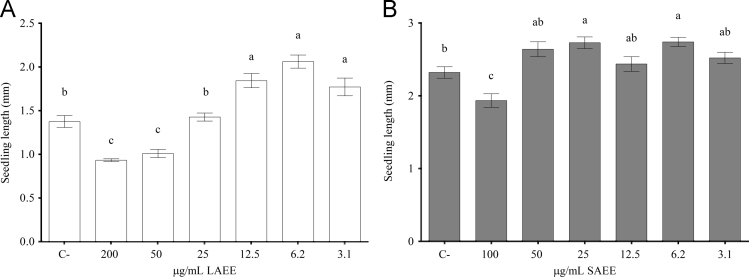
High concentration ranges of ethyl linoleate (LAEE) and ethyl stearate (SAEE) in *Nicotiana tabacum* seedling germination, showing a concentration dependent transition from growth inhibition to growth induction in each compound.

For purification, mass spectrometry and nuclear magnetic resonance (NMR) [2], [3], a modified Folch extraction [4] was standardized as described in [1], (Supplementary Data Set A and Fig. 1A, B). Carbon source effects were then studied in *L. theobromae* using the standardized Folch extraction. Fatty acid ester production was studied in the other fungal species *N. parvum, F. oxysporum* and *T. asperellum* for comparison. All samples, including the positive controls, were analyzed for naturally produced fatty acid ethyl esters by gas chromatography/mass spectrometry (GC–MS) as described in [1] (Fig. 2, Fig. 3, Fig. 4, Fig. 5). The data was expressed as percent yield of each compound from the total compounds identified (Table 1). Morphology of *L. theobromae* incubated in 5% glucose was documented by photography and compared to the morphology in 5% glucose+5% grapeseed oil (Fig. 6). With the aim to test the effect of the isolated compounds *in planta*, we chose tobacco (*Nicotiana tabacum),* a well-studied plant model [5] to measure growth as described in [1]. The length of the seedling was measured after 7–10 days post-sowing using calibrated Image J software [6] from cotyledon tip to root tip for each experimental condition. Morphology was also assessed and documented 45 days post-dosing and sowing (Fig. 7, Fig. 8). A high concentration gradient for the most physiologically active fatty acid esters found and described in [1] was performed by germinating the plant model *N. tabacum* in SAEE from 100 to 3.1 μg/mL and LAEE from 200 to 3.1 μg/mL (Fig. 9).

## Figures and Tables

**Table 1:** Mean area under the curve (AUC) data presented as percent yields of total of the compounds identified in each strain and in each carbon source.

Identified compounds	5% grapeseed oil	5% glucose	5% grape-seed oil	5% glucose+5% grapeseed oil			
	Grapeseed oil Fischer esterification (%)	*L. theobromae* UCD 256 Ma (%)	*L. theobromae* UCD 256 Ma (%)	*L. theobromae* UCD 256 Ma (%)	*N. parvum UCD646So* (%)	*F. oxysporum* (%)	*T. asperellum* (%)

Methyl hexadecanoate	1.9	0.0	2.7	0.0	0.0	0.0	0.0
Ethyl hexadecanoate (PAEE)	2.5	16.8	1.6	6.1	6.0	44.8	2.8
Hexadecanoate, 2-methylpropyl ester	0.0	0.0	0.0	0.0	0.0	0.0	0.0
9-Octadecenoate (Z)- methyl ester	31.2	0.0	44.0	0.7	5.2	0.0	20.9
Octadecanoate ethyl ester (SAEE)	1.0	0.0	0.2	3.9	2.1	4.9	0.0
9-Octadecenoate (Z), ethyl ester (OAEE)	41.9	0.0	0.0	70.4	59.9	15.3	69.5
9-Octadecenoate (E) ethyl ester	0.3	0.0	0.0	0.9	0.9	0.0	0.0
9,12-Octadecadienoate (Z,Z)-, methyl ester	9.2	0.0	9.4	0.1	1.5	0.0	0.0
9,12-Octadecadienoate (Z,Z) ethyl ester (LAEE)	11.7	0.0	40.9	16.2	23.7	35.0	6.8
9,12,15-Octadecatrienoate (Z,Z,Z)-ethyl ester)	0.2	0.0	1.2	1.7	0.7	0.0	0.0
2H-1-Benzopyran, 3,4-dihydro- (R±mellein)	0.0	83.2	0.0	0.0	0.0	0.0	0.0

## Appendix A Supplementary material

Supplementary material

Supplementary material. **Supplementary Data Set A:** High resolution mass spectrometry (HR-MS) of isolated compound from *L. theobromae* incubated in oatmeal indicating a compound with a molecular weight of 308.2.
