# Supplementary material for: Data from mass spectrometry, NMR spectra, GC–MS of fatty acid esters produced by Lasiodiplodia theobromae
Source: Data Brief. 2016 May 9;8:31–9. doi: 10.1016/j.dib.2016.05.003 (PMC4885019; doi:10.1016/j.dib.2016.05.003)
Supplement: Supplementary file 2 — Supplementary material. Supplementary Data Set A: High resolution mass spectrometry (HR-MS) of isolated compound from L. theobromae incubated in oatmeal indicating a compound with a molecular weight of 308.2. [file mmc2.pdf]

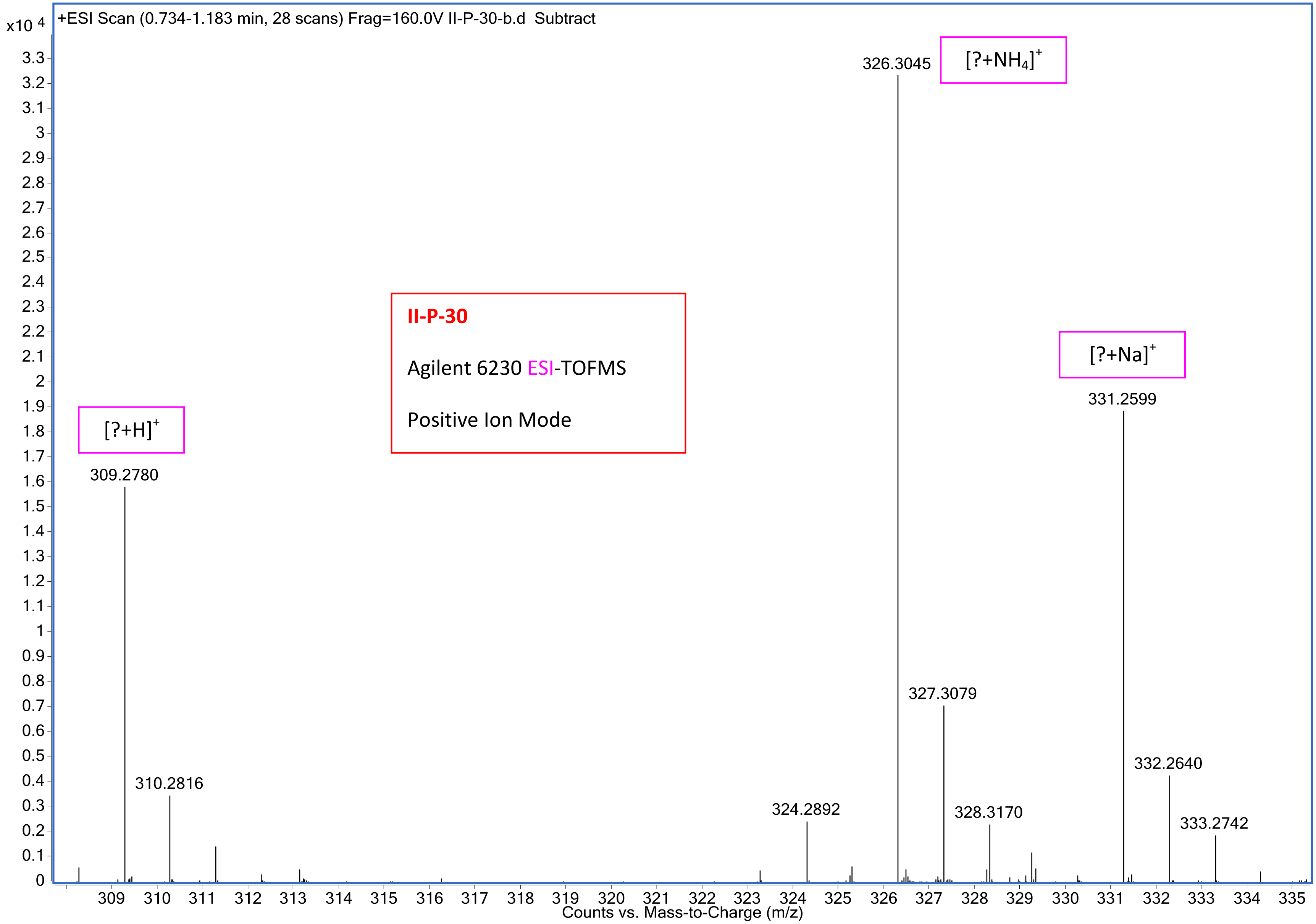

+ESI Scan (0.734-1.183 min, 28 scans) Frag=160.0V II-P-30-b.d Subtract
x10 4
II-P-30
Agilent 6230 ESI-TOFMS
Positive Ion Mode
[?+H]+
309.2780
310.2816
324.2892
326.3045
[?+NH4]+
327.3079
328.3170
[?+Na]+
331.2599
332.2640
333.2742
Counts vs. Mass-to-Charge (m/z)

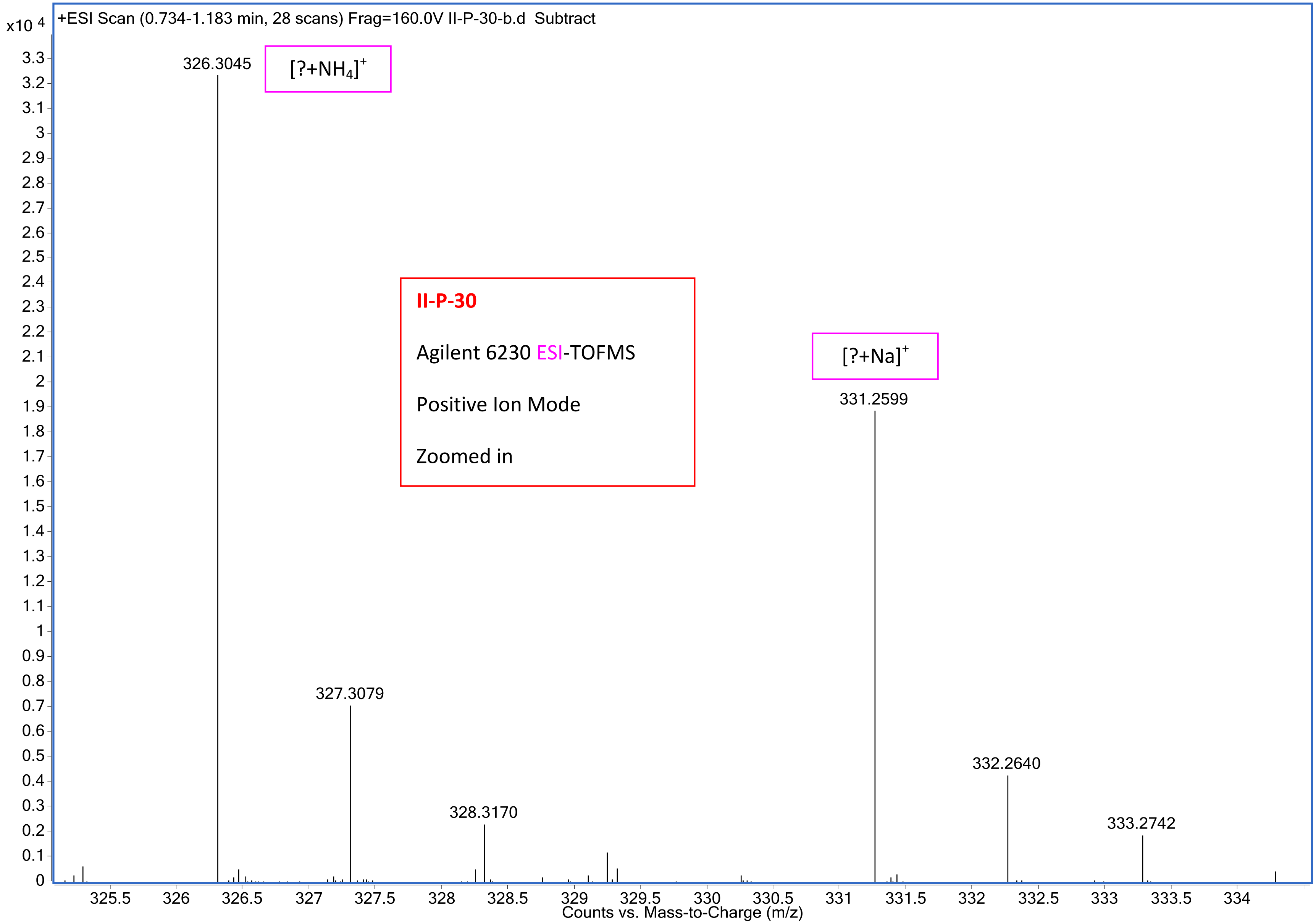
x10 4
+ESI Scan (0.734-1.183 min, 28 scans) Frag=160.0V II-P-30-b.d Subtract
326.3045
[?+NH4]+
II-P-30
Agilent 6230 ESI-TOFMS
Positive Ion Mode
Zoomed in
[?+Na]+
331.2599
327.3079
328.3170
332.2640
333.2742
Counts vs. Mass-to-Charge (m/z)

Search Parameters: Sample II-P-30

	Min#	Max#
C:	5	30
H:	5	60
N:	0	10
O:	0	10
Na:	1	1

Search Results: Sample II-P-30

Mass Measured	Theo. Mass	Delta (ppm)	Composition
331.2599	331.2594	1.5	$[C_{18}H_{34}N_3ONa]^+$
331.2599	331.2608	-2.7	$[C_{20}H_{36}O_2Na]^+$
